# Supplementary material for: The transcriptional landscape and diagnostic potential of long non-coding RNAs in esophageal squamous cell carcinoma
Source: Nat Commun. 2023 Jun 26;14:3799. doi: 10.1038/s41467-023-39530-1 (PMC10293239; doi:10.1038/s41467-023-39530-1)
Supplement: Supplementary file 1 — Supplementary Information [file 41467_2023_39530_MOESM1_ESM.pdf]

Supplementary Information

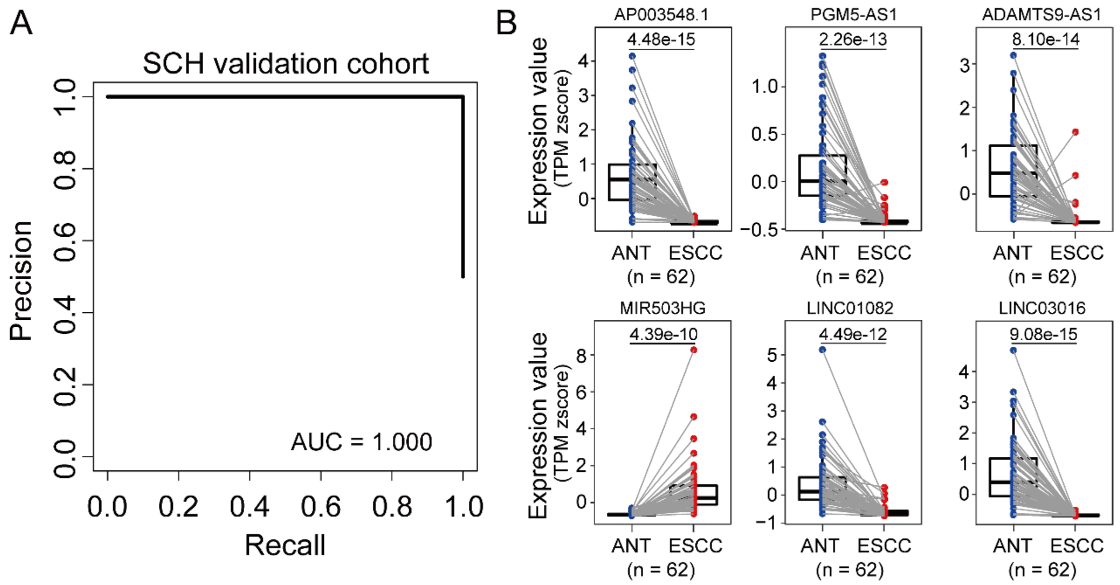

Supplementary Figure S1. Verification of the MLMRPscore in the SCH validation cohort. (A) A precision-recall curve for the performance of the MLMRPscore. (B) Boxplots showing expression levels of six lncRNA biomarkers. For the boxplot, the center line indicates the median; box limits indicate the first and third quartiles; whiskers encompass the 1.5X interquartile range. P values were determined by two-tailed paired t-tests without adjustments for multiple comparisons. ESCC, esophageal squamous cell carcinoma; ANT, adjacent normal tissues; AUC, area under the curve.

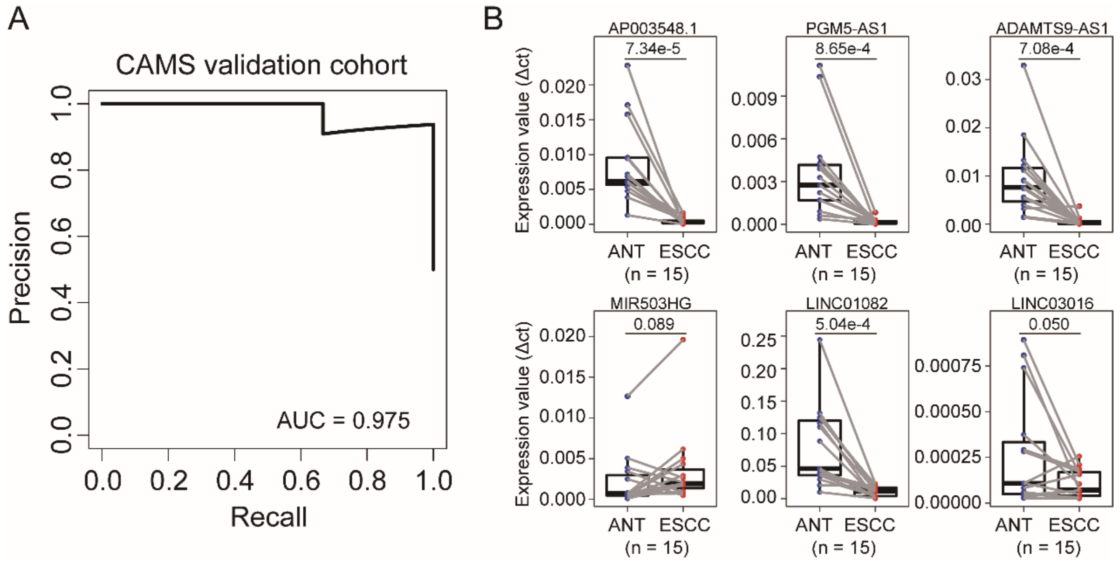

Supplementary Figure S2. Verification of the MLMRPscore in the CAMS validation cohort. (A) A precision-recall curve for the performance of the MLMRPscore. (B) Boxplots showing expression levels of six lncRNA biomarkers. For the boxplot, the center line indicates the median; box limits indicate the first and third quartiles; whiskers encompass the 1.5X interquartile range. P-values were determined by two-tailed paired t-tests without adjustments for multiple comparisons. ESCC, esophageal squamous cell carcinoma; ANT, adjacent normal tissues; AUC, area under the curve.

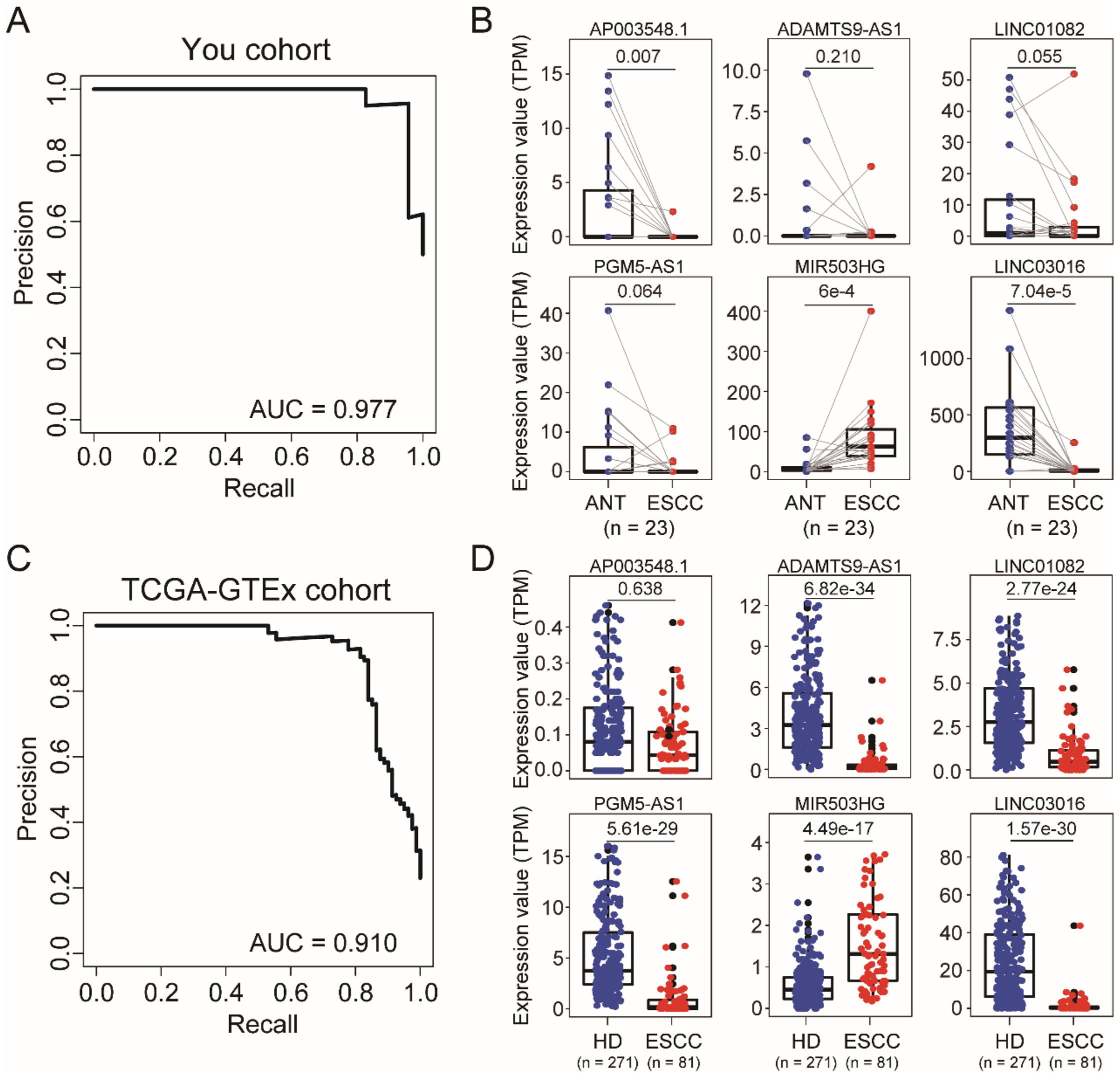

Supplementary Figure S3. Verification of the MLMRPscore in two external RNA-seq cohorts. A precision-recall curve for the performance of the MLMRPscore in the You cohort (A) and TCGA-GTEx cohort (C). Boxplots showing expression levels of six lncRNA biomarkers in the You cohort (B) and TCGA-GTEx cohort (D). For the boxplot, the center line indicates the median; box limits indicate the first and third quartiles; whiskers encompass the 1.5X interquartile range. In (B), p values were determined by two-tailed paired t-tests without adjustments for multiple comparisons. In (D), p values were determined by two-tailed Mann-Whitney U tests without adjustments for multiple comparisons. ESCC, esophageal squamous cell carcinoma; ANT, adjacent normal tissues; AUC, area under the curve; HD, healthy donors.

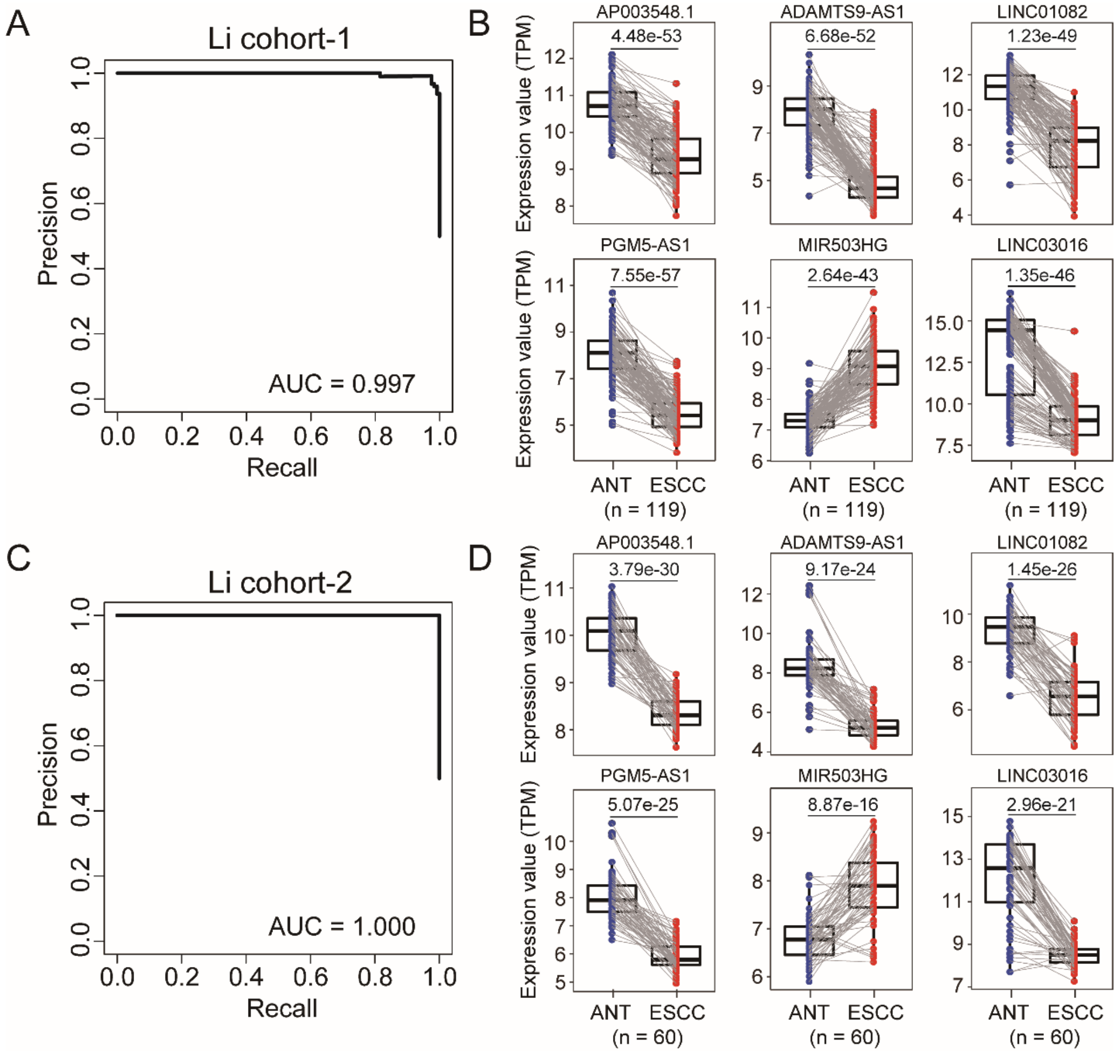

Supplementary Figure S4. Verification of the MLMRPscore in two external Chinese microarray cohorts. A precision-recall curve for the performance of the MLMRPscore in the Li cohort-1 (A) and Li cohort-2 (C). Boxplots showing expression levels of six lncRNA biomarkers in the Li cohort-1 (B) and Li cohort-2 (D). For the boxplot, the center line indicates the median; box limits indicate the first and third quartiles; whiskers encompass the 1.5X interquartile range. In (B, D), p values were determined by two-tailed paired t-tests without adjustments for multiple comparisons. ESCC, esophageal squamous cell carcinoma; ANT, adjacent normal tissues; AUC, area under the curve.

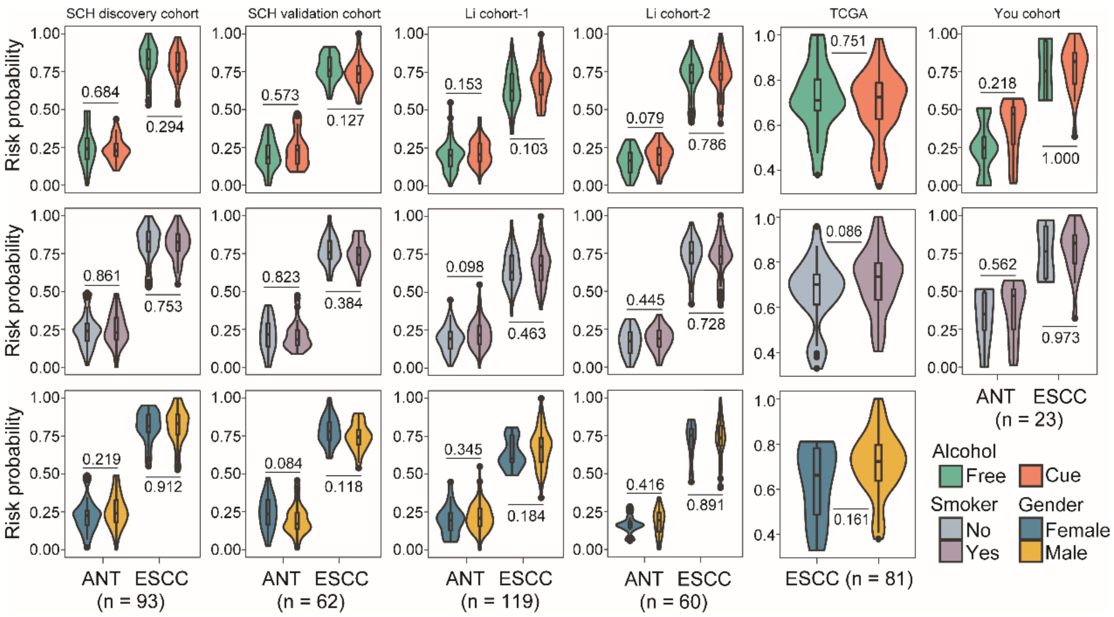

Supplementary Figure S5. Boxplots comparing the MLMRPscore predicted risk probabilities between the two groups with respect to alcohol use, smoking and gender differences in different cohorts. For the boxplot, the center line indicates the median; box limits indicate the first and third quartiles; whiskers encompass the 1.5X interquartile range. P values were determined by two-tailed Mann-Whitney U tests without adjustments for multiple comparisons. ESCC, esophageal squamous cell carcinoma; ANT, adjacent normal tissues; AUC, area under the curve

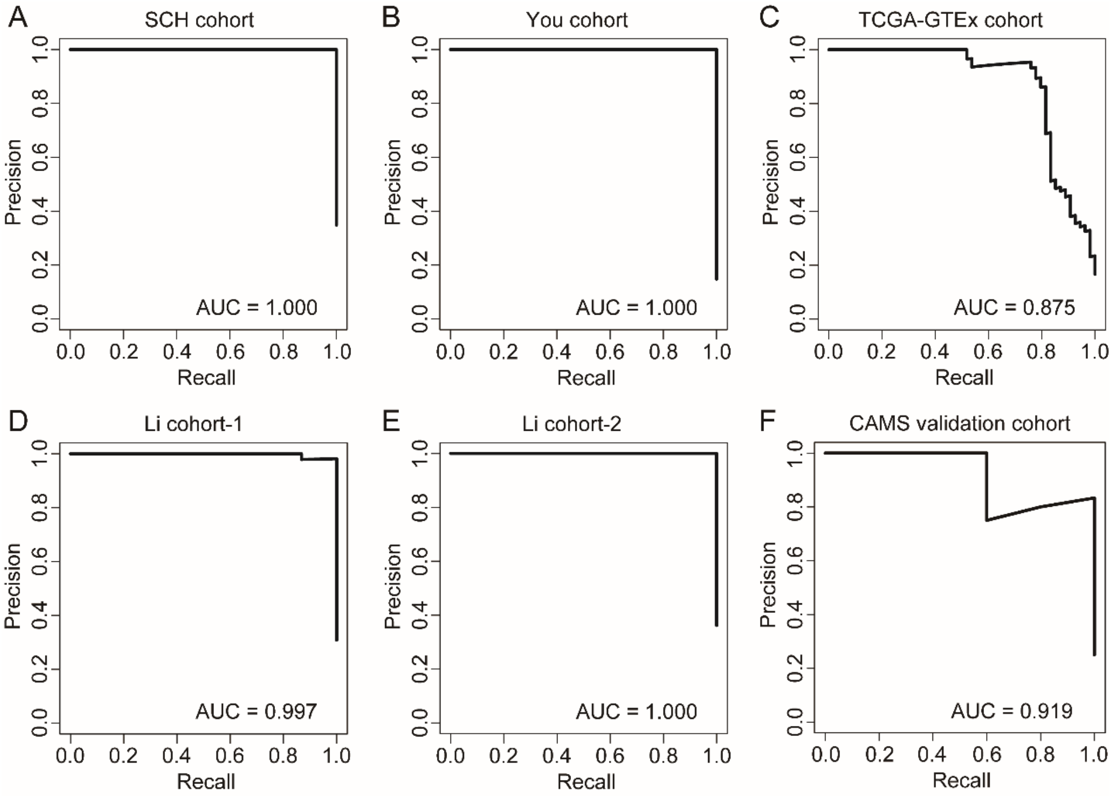

Supplementary Figure S6. A precision-recall curve for the early diagnostic performance of the MLMRPscore in SCH cohort (A), You cohort (B), TCGA-GTEx cohort (C), Li-cohort-1 (D), Li-cohort-2 (E) and CAMS validation cohort (F).
