# Supplementary material for: The transcriptional landscape and diagnostic potential of long non-coding RNAs in esophageal squamous cell carcinoma
Source: Nat Commun. 2023 Jun 26;14:3799. doi: 10.1038/s41467-023-39530-1 (PMC10293239; doi:10.1038/s41467-023-39530-1)
Supplement: Supplementary file 3 — Reporting Summary [file 41467_2023_39530_MOESM3_ESM.pdf]

nature portfolio

Corresponding author(s): Zhihua Liu, Hongyan Chen, Jianzhong Su

Last updated by author(s): May 22, 2023

Reporting Summary

Nature Portfolio wishes to improve the reproducibility of the work that we publish. This form provides structure for consistency and transparency in reporting. For further information on Nature Portfolio policies, see our Editorial Policies and the Editorial Policy Checklist.

Statistics

For all statistical analyses, confirm that the following items are present in the figure legend, table legend, main text, or Methods section.

n/a	Confirmed	
☐	☒	The exact sample size (n) for each experimental group/condition, given as a discrete number and unit of measurement
☐	☒	A statement on whether measurements were taken from distinct samples or whether the same sample was measured repeatedly
☐	☒	The statistical test(s) used AND whether they are one- or two-sided<br>*Only common tests should be described solely by name; describe more complex techniques in the Methods section.*
☒	☐	A description of all covariates tested
☒	☐	A description of any assumptions or corrections, such as tests of normality and adjustment for multiple comparisons
☐	☒	A full description of the statistical parameters including central tendency (e.g. means) or other basic estimates (e.g. regression coefficient) AND variation (e.g. standard deviation) or associated estimates of uncertainty (e.g. confidence intervals)
☐	☒	For null hypothesis testing, the test statistic (e.g. F, t, r) with confidence intervals, effect sizes, degrees of freedom and P value noted<br>*Give P values as exact values whenever suitable.*
☒	☐	For Bayesian analysis, information on the choice of priors and Markov chain Monte Carlo settings
☒	☐	For hierarchical and complex designs, identification of the appropriate level for tests and full reporting of outcomes
☒	☐	Estimates of effect sizes (e.g. Cohen's d, Pearson's r), indicating how they were calculated

Our web collection on statistics for biologists contains articles on many of the points above.

Software and code

Policy information about availability of computer code

Data collection	No software was used for data collection.
Data analysis	STAR-2.6.1 was used for RNA alignment. HTSeq 0.13.5 was used to calculate transcript abundances at the gene level. Bowtie2 v2.3.4.2 was used to map probe sequences to the human reference genome (GRCh38/hg38). DESeq2 1.34.0 was used to perform differential gene expression analysis. TDM 0.3 was used to normalize RNA-seq data to make them comparable to microarray data for use with machine learning. Caret 6.0-92 was used to perform feature selection (RF-RPE algorithm) and classification. Pheatmap 1.0.12 was used to conduct unsupervised clustering. ClusterProfiler 4.2.2 was used to perform KEGG pathway enrichment. PROC 1.18.0 was used to measure the performance of a classification model. OptimalCutpoints 1.1-5 as used to find a optimal cutoff point for a binary classifier.<br>The source code of this work is available at GitHub (https://github.com/ZhouSunLab-Workshops/MLMRP).

For manuscripts utilizing custom algorithms or software that are central to the research but not yet described in published literature, software must be made available to editors and reviewers. We strongly encourage code deposition in a community repository (e.g. GitHub). See the Nature Portfolio guidelines for submitting code & software for further information.

Data

Policy information about availability of data

All manuscripts must include a data availability statement. This statement should provide the following information, where applicable:

- Accession codes, unique identifiers, or web links for publicly available datasets
- A description of any restrictions on data availability
- For clinical datasets or third party data, please ensure that the statement adheres to our policy

LncRNA expression profiles generated during this study are provided in Supplementary Data 1. All public ESCC data are available from the Gene Expression Omnibus (GEO) database under accession number GSE53624 (www.ncbi.nlm.nih.gov/geo/query/acc.cgi?acc=GSE53624), GSE53622 (www.ncbi.nlm.nih.gov/geo/query/acc.cgi?acc=GSE53622) and GSE130078 (www.ncbi.nlm.nih.gov/geo/query/acc.cgi?acc=GSE130078) and UCSC Xena (https://xenabrowser.net/datapages/?cohort=GDC%20TCGA%20Esophageal%20Cancer%20(ESCA)&removeHub=https%3A%2F%2Fxena.treehouse.gi.ucsc.edu%3A443).

Human research participants

Policy information about studies involving human research participants and Sex and Gender in Research.

Reporting on sex and gender	Among 155 patients, the number of male and female patients is 103 and 52, respectively. The ratio is about 2:1, which is consistent with the epidemiological characteristic of ESCC.
Population characteristics	1. SCH cohorts: 155 patients with ESCC (103 males and 53 females), Median age: 62, Number of patients at stage I/II: 83, Number of patients with smoking history: 68, Number of patients with drinking history: 49.<br>2. CAMS tissue cohort: 15 patients with ESCC, Median age: 60, Number of patients at stage I/II: 5.<br>3. CAMS plasma cohort: 32 healthy controls (4 females and 28 males, Median age: 62), 13 patients with esophageal intraepithelial neoplasia (3 females and 10 males, Median ages: 56), 32 patients with ESCC (5 males and 27 males, Median age: 62.5).<br>4. Li cohort-1: 119 patients with ESCC (21 females and 98 males), Median age: 59, Number of patients at stage I/II: 53, Number of patients with smoking history: 80, Number of patients with drinking history: 74.<br>5. Li cohort-2: 60 patients with ESCC (12 females and 48 males), Median age: 60.5, Number of patients at stage I/II: 34, Number of patients with smoking history: 34, Number of patients with drinking history: 32.<br>6. You cohort: 23 patients with ESCC (2 females and 21 males), Number of patients at stage I/II: 4, Number of patients with smoking history: 17, Number of patients with drinking history: 19.<br>7. TCGA cohort: 81 patients with ESCC (12 females and 69 males), Median age: 57, Number of patients at stage I/II: 54 (2 without stage information), Number of patients with smoking history: 51 (3 without smoking information), Number of patients with drinking history: 60 (2 without drinking information).<br>8. GTEx cohort: 271 Esophagus Mucosa samples.
Recruitment	1. SCH cohorts: A total of 155 patients diagnosed with ESCC between May 2017 and July 2018 at Shanxi Cancer Hospital, China, were recruited to participate consecutively in this study. None of the patients had received prior treatment for their disease, and tumors were classified based on WHO criteria. Since specimens were acquired and stored under the same conditions, and processing and data acquisition under the same conditions, no bias appeared.<br>2. CAMS tissue cohort: A total of 15 patients diagnosed with ESCC were recruited consecutively. None of the patients had received prior treatment for their disease, and tumors were classified based on WHO criteria. Since specimens were acquired and stored under the same conditions, and processing and data acquisition under the same conditions, no bias appeared.<br>3. CAMS plasma cohort: 45 patients diagnosed with ESCC or esophageal intraepithelial neoplasia and 32 healthy volunteers between Jan 2023 and Mar 2023 were recruited consecutively and simultaneously in this cohort. None of the patients had received prior treatment for their disease, and tumors were classified based on WHO criteria. Since blood took, plasma separated and stored for each sample under the same conditions, and RNA extraction and RT-qPCR detections were performed under the same conditions, no bias appeared.
Ethics oversight	This study was performed according to the Declaration of Helsinki and approved by the Ethics Committee of the Cancer Hospital of the Chinese Academy of Medical Sciences (CAMS) and the Shanxi Medical University and Shanxi Cancer Hospital. Informed consent was obtained from all subjects, and all data were anonymously analyzed.

Note that full information on the approval of the study protocol must also be provided in the manuscript.

Field-specific reporting

Please select the one below that is the best fit for your research. If you are not sure, read the appropriate sections before making your selection.

☒ Life sciences ☐ Behavioural & social sciences ☐ Ecological, evolutionary & environmental sciences

For a reference copy of the document with all sections, see nature.com/documents/nr-reporting-summary-flat.pdf

Life sciences study design

All studies must disclose on these points even when the disclosure is negative.

Sample size	1. SCH cohorts: A total of 155 patients diagnosed with ESCC between May 2017 and July 2018 at Shanxi Cancer Hospital, China, were recruited to participate consecutively in this study.<br>2. CAMS tissue cohort: A total of 15 patients diagnosed with ESCC were recruited consecutively.<br>3. CAMS plasma cohort: 45 patients diagnosed with ESCC or esophageal intraepithelial neoplasia and 32 healthy volunteers between Jan 2023 and Mar 2023 were recruited consecutively and simultaneously in this cohort.
Data exclusions	Since inclusion criteria were strictly enforced and the data qualities were all qualified, no data were excluded.
Replication	As for each patient, there is no replication. As for diagnosis model, we tested its efficiency in 3 in-house cohorts and 4 public cohorts.
Randomization	This design is not relevant to the study; No experimental intervention requiring randomization was applied.
Blinding	The participants were not blinded to data collection and analysis since the study is non interventional retrospective study.

Reporting for specific materials, systems and methods

We require information from authors about some types of materials, experimental systems and methods used in many studies. Here, indicate whether each material, system or method listed is relevant to your study. If you are not sure if a list item applies to your research, read the appropriate section before selecting a response.

Materials & experimental systems

n/a	Involved in the study	
☒	☐	Antibodies
☒	☐	Eukaryotic cell lines
☒	☐	Palaeontology and archaeology
☒	☐	Animals and other organisms
☒	☐	Clinical data
☒	☐	Dual use research of concern

Methods

n/a	Involved in the study	
☒	☐	ChIP-seq
☒	☐	Flow cytometry
☒	☐	MRI-based neuroimaging
