# Supplementary material for: The transcriptional landscape and diagnostic potential of long non-coding RNAs in esophageal squamous cell carcinoma
Source: Nat Commun. 2023 Jun 26;14:3799. doi: 10.1038/s41467-023-39530-1 (PMC10293239; doi:10.1038/s41467-023-39530-1)
Supplement: Supplementary file 4 — Description of Additional Supplementary Files [file 41467_2023_39530_MOESM4_ESM.pdf]

Description of Additional Supplementary Files

File Name: Supplementary Data 1

Description: Lists of significant differentially expressed lncRNAs.

File Name: Supplementary Data 2

Description: Lists of six lncRNA biomarkers.

File Name: Supplementary Data 3

Description: Clinical information and features of 15 ESCC patients from the CAMS cohort.

File Name: Supplementary Data 4

Description: Clinical information and expression levels of six lncRNA biomarkers of 32 ESCC patients, 32 healthy controls, and 13 EIN patients from the CAMS plasma cohort.

File Name: Supplementary Data 5

Description: Expression profiles of 16,064 lncRNAs of paired tumor and non-cancerous tissues from 155 ESCC patients at SCH.

File Name: Supplementary Data 6

Description: Primers sequences of six lncRNA biomarkers.
